# The Effects of Enriched Rehabilitation on Cognitive Function and Serum Glutamate Levels Post-stroke

**DOI:** 10.3389/fneur.2022.829090

**Published:** 2022-03-17

**Authors:** Xin Wang, Yuan Peng, Hongyu Zhou, Wanchun Du, Junya Wang, JiaJin Wang, Tong Wu, Xiaojia Tang, Yichen Lv, Jianwei Gong

**Affiliations:** ^1^Department of Rehabilitation Medicine, Clinical Medical College, Yangzhou University, Yangzhou, China; ^2^Department of Rehabilitation Medicine, Guangzhou First People's Hospital, School of Medicine, South China University of Technology, Guangzhou, China; ^3^Medical College, Yangzhou University, Yangzhou, China; ^4^Department of Rehabilitation Medicine, Yangzhou Clinical Medical College of Dalian Medical University, Yangzhou, China; ^5^School of Rehabilitation Medicine, Binzhou Medical University, Yantai, China

**Keywords:** enriched rehabilitation, cognitive function, stroke, glutamate, synaptic plasticity, oxidative stress

## Abstract

**Aim:**

This study aimed to explore the effect of enriched rehabilitation (ER) on cognitive function and serum glutamate levels in patients with stroke.

**Methods:**

Forty patients diagnosed with post-stroke cognitive impairment (PSCI), according to the inclusion criteria, and undergoing inpatient rehabilitation were enrolled in the study. Patients were randomly assigned to receive 8 weeks of ER treatment (ER group; *n* = 20) or conventional medical treatment (CM group; *n* = 20). In addition, 20 age-matched healthy subjects who were outpatients in our hospital during the same period formed the healthy control (HC) group. In- and between-group differences in cognitive function were assessed during pre-intervention and post-intervention based on the Montreal Cognitive Assessment (MoCA), the Symbol Digit Modalities Test (SDMT), and the Trail Making Test (TMT). The serum levels of glutamate, tumor necrosis factor (TNF), and malondialdehyde (MDA) levels were also detected pre-intervention and post-intervention.

**Results:**

Pre-intervention cognitive function and the levels of all the serum parameters assessed significant difference between the HC group and the PSCI group (both ER and CM groups) (*p* < 0.05), but not between the two groups of patients with PSCI (*p* > 0.05). Significant improvements were observed in cognitive function in both the ER and the CM groups post-intervention compared with pre-intervention, as evidenced by the measured improvement in MoCA, SDMT, and TMT scores. Similar improvements were seen for serum glutamate, the degree of oxidative damage, and the level of inflammation in both the treatment groups (*p* < 0.05). More enhancements in cognitive function, including MoCA, SDMT, TMT scores, and the serum levels of glutamate, the degree of oxidative damage, and the level of inflammation were shown in the ER group compared with the CM group post-intervention (*p* < 0.05).

**Conclusions:**

ER can improve cognitive function in patients with PSCI. The associated mechanism may be related to the negative regulatory effect of ER on serum glutamate, TNF, and MDA levels, which is likely to enhance synaptic plasticity and alleviate oxidative stress- and inflammation-related damage, at least to some extent.

## Introduction

Post-stroke cognitive impairment (PSCI) is one of the most common sequelae of stroke. Over 70% of survivors have cognitive impairment in the first week post-stroke while 37% still display cognitive deficits after 6 months (1). PSCI is strongly associated with reduced quality of life and long-term survival (2–4). Pharmacological therapeutic strategies, such as the use of cholinesterase inhibitors, have shown only limited efficacy against PSCI and many side effects, highlighting the need to explore and identify non-pharmacological strategies for the treatment of this condition (4).

Enriched rehabilitation (ER), a comprehensive strategy that combines environmental enrichment and task-oriented exercises, has shown the potential to improve cognitive function in animal models of central nervous system diseases, including stroke and Parkinson's disease (5, 6). Several recent clinical studies have also demonstrated that early ER intervention in the stroke unit can effectively improve motor function after stroke (7–9). However, little is known about the effects of ER on cognitive function in PSCI or about the underlying mechanisms.

Neurotransmitters are critical in cognitive function. Glutamate is an excitatory neurotransmitter that has been implicated in cognitive function, especially in memory and learning processes. After a stroke, glutamate levels in the infarcted site were found to be up to 80-fold higher compared with its physiological level, resulting in elevated glutamate contents in the cerebrospinal fluid and blood (10). However, excessive glutamate can lead to synaptic excitotoxicity, and even neuronal death, through aggravating oxidative stress and inflammation (11, 12). Some studies have shown that high serum glutamate levels are correlated with poor cognitive outcomes (11, 13), while others have reported that physical therapy can decrease serum glutamate contents to some extent and is associated with an improvement in overall function (14). In addition, chronic neuroinflammation is an inevitable response post-stroke, which is related to functional outcomes. Tumor necrosis factor (TNF), one of the key components in neuroinflammation secreted by activated microglia, is verified to be associated with cognitive impairment. Besides, oxidative stress, mediated by reactive oxygen species (ROS)-induced damage, plays a devastating role in stroke pathogenesis. Malondialdehyde (MDA) would accelerate the chain reaction of ROS formation and is used as a biomarker of the oxidative stress level corelated with cognitive function (15).

Given that ER has been associated with improved cognitive outcome brain functions and that serum glutamate levels have been associated with cognition, in this study, we explored the effect of ER on cognitive function, serum glutamate levels, oxidative stress responses, and inflammation-related responses in patients with PSCI.

## Materials and Methods

### Participants

All patients with cerebral infarction at the Yangzhou University Clinical School from January 2020 to June 2021 were assessed for enrollment in the study based on the following inclusion criteria: cerebral infarction confirmed by MRI scan due to the lesion in the internal carotid artery system; meeting the diagnostic criteria for PSCI with a Montreal Cognitive Assessment (MoCA) score between 18 and 23; the absence of sensory aphasia and having the ability to communicate with others, at least simply; primary and unilateral onset and a volume of ischemic necrosis between 20 and 40 ml as determined diffusion-weighted imaging (DWI) (13) (how the infarct volume was calculated is described in Supplementary Materials); 55–70 years of age; time from onset between 2 and 3 months; Brunnstrom Stages of the affected limbs (upper and lower) between grade IV and V and capable of intracommunity walking with assistance; education level greater than high school degree (>12 years of education); stable vital signs; and capable of completing 8 weeks of rehabilitation training as required. The exact exclusion criteria are listed in Supplementary Materials.

Among the 217 identified patients, 61 met the inclusion criteria. Of these, 12 were unwilling to participate in the study. As measured by the clinical sample size, a minimum of 20 participants was required for each group (how to measure sample size is described in Supplementary Materials). Forty of the remaining 49 patients were assigned either to an ER group or a conventional medical treatment (CM) group according to a random number table, with 20 patients assigned to each group. The patients were blind to the grouping. We marked each group with letters and numbers, and the patients did not know the specific grouping situation. In the follow-up study, the therapists were assigned individual tasks without knowing the specific groups of patients. Twenty age-matched healthy subjects from the outpatient department comprised the healthy control (HC) group. The termination criteria of the study were (1) a participant presenting with serious adverse events and deemed to be incapable of continuing the trial and (2) a participant withdrawing from the trial.

**Figure 1:**
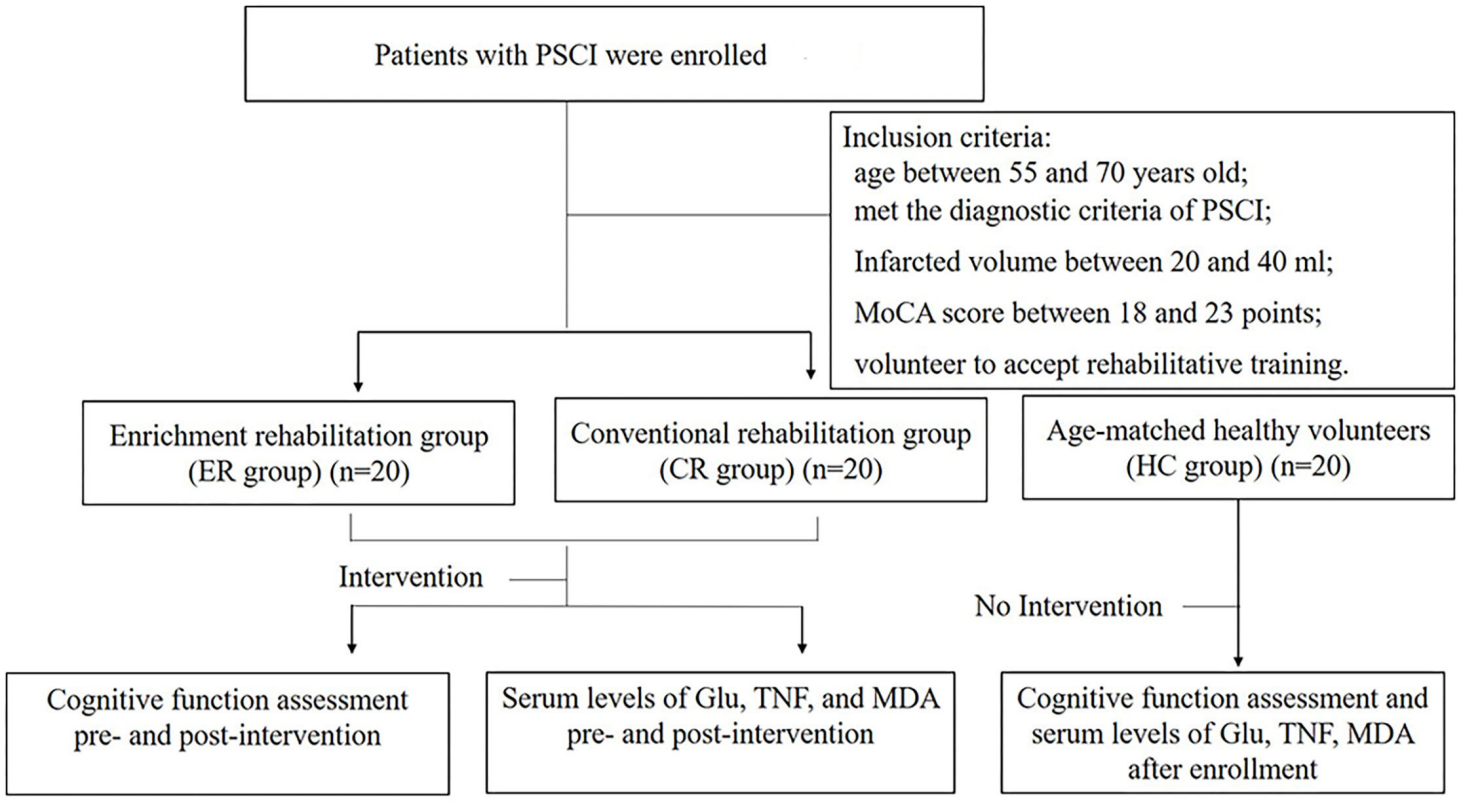
Flowchart illustrating the process of the study.

This study was approved by the Medical Ethics Committee of the Clinical Medical College of Yangzhou University (Ethical Approval No. 2016055). All patients signed an informed consent form. The general process of the study is shown in Figure 1.

### Intervention

The patients in the CM group received conventional medication, including drugs for controlling blood pressure, lowering cholesterol, improving cerebral blood flow (nimodipine 20 mg two times a day), promoting cognitive function (huperzine A 100 mg twice a day), and other related medications, according to Chinese Guidelines for diagnosis and treatment of acute ischemic stroke 2018 and other guidelines for diagnosis and treatment of ischemic stroke (10, 16, 17). In the ER group, treatment was administered for 8 consecutive weeks (2 h each time, once a day, 6 days per week), in addition to conventional medication (9, 18–21). The patients in the ER group completed various enrichment activities for 2 h in specific places using computers with an internet connection, virtual reality technology, as well as other equipment, as described below. All rehabilitative activities were of mild to moderate intensity and were monitored by a wristwatch (Apple Watch Series5) during the rehabilitation sessions, which induced a target heart rate below 65–70% of the maximum heart rate (22, 23).

#### Sensory-Motor Stimulation

##### Visual Stimulation

The Internet was used to select the reading materials and images the patients were interested in for reading and viewing one time a day for 10 min each time.

##### Olfactory Stimulation

The patients smelled two bottles of perfume with different scents and tried to name the scents for 5 min each time.

##### Tactile Stimulation

Toys or objects of different shapes, sizes, and textures were placed in black cotton cloth bags such that patients could not see the objects. The patients tried to name the objects or describe their characteristics by touching them with the hand on the paralyzed side. This process lasted for 10 min each time.

##### Exercise Stimulation

For lower limb exercise, the patients performed activities such as field walking, mushroom picking in the forest, or skiing in the snow using virtual reality equipment and a flat treadmill. For upper limb exercise, the patients were asked to perform tasks such as painting with a brush or making different items with plasticine. The patients were required to use the paralyzed limb aided by the healthy limb. The task lasted for 15 min each time.

#### Cognitive Activities

Patients in the ER group were required to complete specific tasks by integrating multiple cognitive functions using virtual reality equipment or actual scenarios, including:

##### Supermarket Shopping

The patients were given a list before shopping and had to return the list within a specified time. The patients had to purchase the listed items in the supermarket by themselves.

##### Classroom Observation

Patients had to attend lectures in the room and to recite the content as much as possible, just like student activity. Stimuli such as noise, birds, and other people's voices were added to simulate interference during the class. Therapists asked questions after the class.

##### Card Games

A set of cards with different colors and graphics was used. The therapist placed three cards according to a specific rule (graphics, color, or number) and asked the patient to identify the rule and select the appropriate fourth card.

##### Taking the Subway

This task simulated a patient going home from the hospital. The patient was asked to complete planning the route, to choose a station to board and disembark, to enter and exit subway stations on their own, etc.

Cognitive activities were performed one time daily for a total of 40 min.

#### Social Activities

Participants were encouraged to communicate with each other during the entire ER training process; to play card games, chess, table tennis, and other sports involving more than one person; and to express their opinions on a hot social topic.

Social activities lasted for 40 min each time. All the above activities were undertaken under the guidance of therapists.

### Outcomes and Measurement Procedures

All the patients completed the assessment of cognitive function and the measurement of serum parameters within 48 h before the first treatment and 48 h after the 8-week rehabilitation process, respectively. The HC group completed the above tests within 24 h after enrollment.

#### Evaluation of Cognitive Function

The overall cognitive function of patients in each group was determined based on the MoCA score. The assessment comprised eight items, including visual space and executive function, attention, naming, memory, abstract reasoning, language, delayed recall, and orientation ability, up to a maximum of 30 points, with ≥26 points being considered normal (24).

The symbol digit modalities test (SDMT) was used to assess attentional function. The SDMT records the number correctly entered within 90 s as the final score, excluding the number filled in during the practice (25).

The Trail Making Test (TMT) was used to evaluate the executive function, in which part A (TMT-A) is connected in sequence from numbers 1–25. TMT part B (TMT-B) is connected sequentially with numbers and letters alternating. The time spent recording TMT-A and TMT-B represented the evaluation index (26).

#### Serum Glutamate Level

All the patients were assessed for serum glutamate levels within 48 h before treatment, within 48 h after 8 weeks of treatment, and 12 h after fasting. In the morning, blood was drawn into a test tube without anticoagulant. Once it had precipitated at room temperature, the serum was collected and stored at −80°C. The glutamate concentration in serum was detected by high-performance liquid chromatography (13). The experimental procedure is as described in our previously published article (12). Glutamate concentration in the serum was calculated according to the standard curve (Amino acid standard reagent: Sigma, USA. Catalog number: AAS18-5ML). The concentrations of serum glutamate were expressed as μmol/L.

#### Serum MDA Level

The serum MDA level is a sensitive index reflecting the degree of oxidative damage in the body (27). Serum was collected as described above. The concentration of serum MDA was estimated according to the method using thiobarbituric acid reagent, and the absorbance of the supernatant was measured spectrophotometrically at 530 nm (28) (MDA Test Kit: Comin Biotechnology Co. Ltd., China. Catalog numbers: MDA-1-Y). The concentrations of MDA were expressed as μmol/L.

#### Serum TNF Level

The serum TNF content is a sensitive indicator that reflects the level of inflammation in the body (29). Serum was collected as described for the determination of glutamate content. The serum TNF concentration was determined by ELISA [(30); TNF ELISA Kit: Elabscience Biotechnology Co., Ltd., China. Catalog numbers: E-EL-H0109c]. All the measurements were performed according to the manufacturer's instructions. Briefly, the standard series was made by producing a series of diluted concentrations: 500, 250, 125, 61.5, 31.2, 15.6, and 7.8 pg/ml TNF to compare the differences in the gained absorbance. For the preparation of the standard addition series, we have diluted the 500 pg/ml standard 1:1 with blood serum from patients or healthy controls, resulting in 250 pg/ml recombinant human TNF standard that additionally contains extra TNF from the blood serum. Then, the absorbance at 450 nm was detected on the ELISA microplate reader (Spectra Max 190, Molecular Devices) for calculating the TNF level. The concentrations of TNF were expressed as pg/ml.

### Statistical Analysis

Data were analyzed using SPSS 24.0 (IBM Corp., Armonk, NY, USA). Results are presented as means ± standard deviation (SD) or median (quartiles) for continuous variables, depending on the normal or non-normal distribution of data. It was assessed to the normality of data by Shapiro–Wilk, and the variance homogeneity of data by one-way ANOVA test/Levene test. A chi-square test was used to analyze the between-group differences for categorical variables, such as gender and stroke site. A one-way, repeated-measures ANOVA or the Kruskal–Wallis test was performed to detect between-group differences (ER, CM, and HC groups) for continuous variables, which included MoCA, SDMT, and TMT scores as well as serum levels of glutamate, TNF, and MDA pre-intervention and post-intervention. A paired t-test or the Wilcoxon Rank Checking test was used to detect within-group differences (pre-intervention vs. post-intervention in the PSCI groups) for continuous variables. A least-significant difference test or the Mann–Whitney *U* test was utilized to compare the between-group differences post-intervention (ER and CM groups) for continuous variables, including MoCA, SDMT, and TMT scores and serum levels of glutamate, TNF, and MDA post-intervention. The significance was set at *p* < 0.05.

## Results

### Basic Demographic Information

In this study, the data including the scores of MoCA and TMT-A, and the serum concentration of TNF were not normally distributed nor was variance homogeneity met. There were 10 men and 10 women in the CM group, with an average age of 60.95 ± 5.70 years. The ER group consisted of 9 men and 11 women, with an average age of 61.70 ± 5.59 years. The HC group comprised 11 men and 9 women, with an average age of 61.03 ± 5.42 years. The baseline information for each group is presented in Table 1.

**Table 1:** Comparison of the basic demographic information for each group.

	**HC group (*n* = 20)**	**CM group (*n* = 20)**	**ER group (*n* = 20)**	***P***
Age (years)	61.03 ± 5.42	60.95 ± 5.70	61.70 ± 5.59	>0.05
Sex (male/female) (*n*)	11/9	10/10	9/11	>0.05
Time since onset (months)		2.46 ± 0.29	2.47 ± 0.31	>0.05
Stroke site (left/right) (*n*)		16/4	15/5	>0.05
Education (years)	14.22 ± 2.03	13.81 ± 1.89	13.75 ± 1.85	>0.05
MoCA		20.12 ± 1.58	19.95 ± 1.66	>0.05
Brunnstrom stage of the affected lower limb (IV/V) (*n*)		9/11	10/10	>0.05
Brunnstrom rating of the affected upper limb (IV/V) (*n*)		10/10	11/9	>0.05
Barthel index		69.05 ± 5.11	68.85 ± 4.73	>0.05
ASL (U/L)		30.02 ± 7.78	29.77 ± 8.28	>0.05
AST (U/L)		33.41 ± 6.98	31.11 ± 6.75	>0.05

*n, number of cases; HC, healthy control; CM, conventional medical treatment; ER, enriched rehabilitation treatment; MoCA, Montreal Cognitive Assessment; ASL, alanine aminotransferase; AST, aspartate aminotransferase*.

### ER Enhanced Cognitive Function in Patients With PSCI

**Figure 2:**
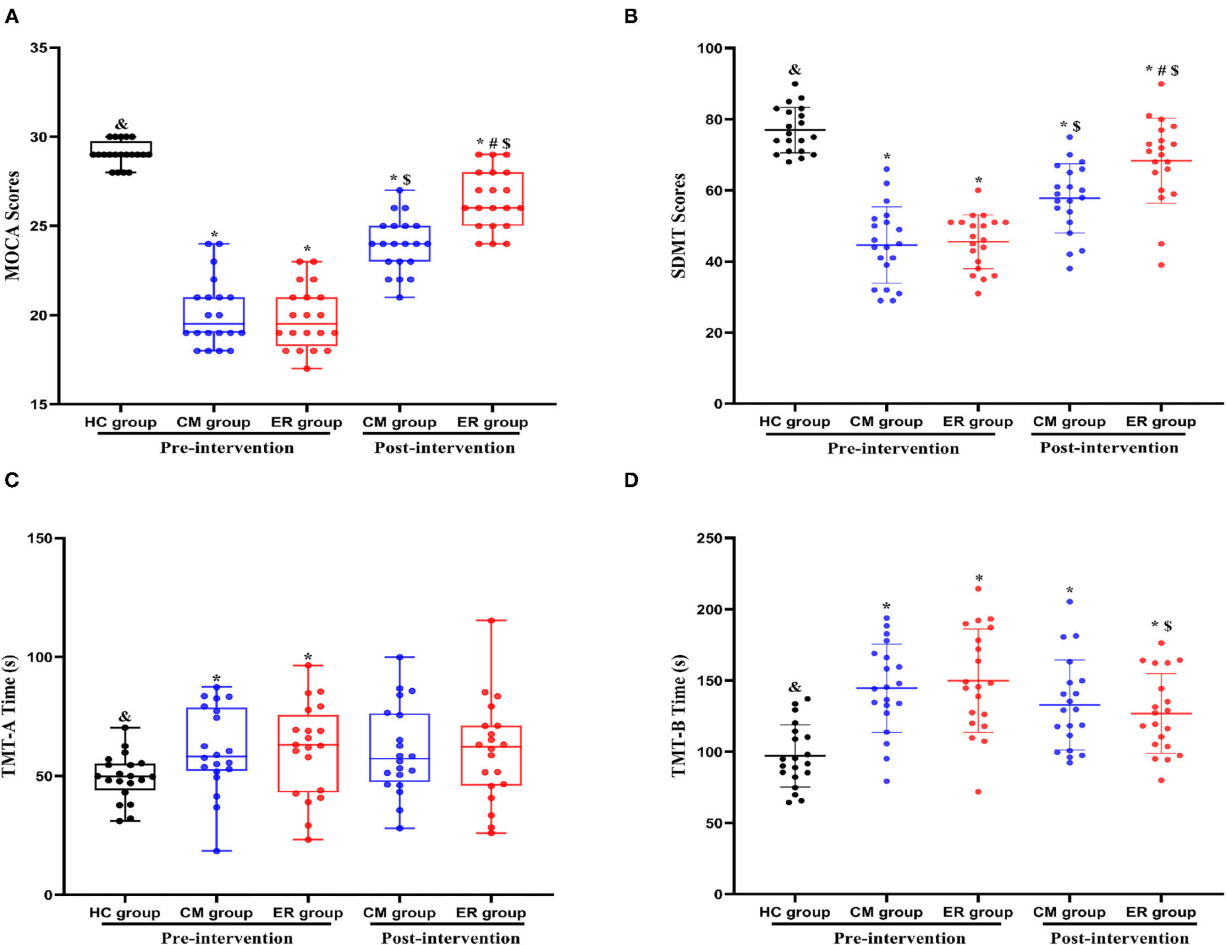
Indexes of cognitive function in each group pre-intervention and post-intervention. *Compared with the HC group, *p* < 0.05. ^&^Compared with the CM group pre-intervention, *p* < 0.05. ^#^Compared with the CM group post-intervention, *p* < 0.05. ^$^Compared with the same group pre-intervention, *p* < 0.05. HC, healthy control; CM, conventional medical treatment; ER, enriched rehabilitation treatment; MoCA, Montreal Cognitive Assessment; SDMT, Symbol Digit Modalities Test; TMT-A, Trail Making Test part A; TMT-B, Trail Making Test part B. **(A)** The scores of MOCA test pre-intervention and post-intervention; **(B)** the scores of SDMT test pre-intervention and post-intervention; **(C)** the time consumption of TMT-A pre-intervention and post-intervention; **(D)** the time consumption of TMT-B pre-intervention and post-intervention.

As shown in Table 2 and Figure 2, significant differences in overall cognitive function, attentional function, and executive function pre-intervention (*p* < 0.05) were found between the HC group and patients with PSCI (both the CM and ER groups) but not between the two groups of patients with PSCI (CM and ER groups). After the intervention, general cognitive function, the attentional function score, and the executive function score of patients in the ER group and the general cognitive function and the attentional function score of patients in the CM group were significantly changed as compared with those before intervention (*p* < 0.05); however, those cognitive function indicators still differed significantly between the HC group and the PSCI group (both the CM and ER groups) (*p* < 0.05). Additionally, the overall cognitive function and attentional function scores were significantly higher in the ER group than in the CM group after intervention (*p* < 0.05).

**Table 2:** Indexes of cognitive function in each group during pre-intervention and post-intervention.

	**HC group**	**CM group**	**ER group**	**F/χ^2^**	***P***
	**(*n* = 20)**	**(*n* = 20)**	**(*n* = 20)**		
**MoCA**					
Pre-intervention	29(28~29.25)^&^	19.5(18~21)^*^	19.5(17~21)^*^	40.12	<0.01
Post-intervention	–	24(21~25)^*^^$^	26(24~28)^*^, *^#^, ^$^*	42.23	<0.01
**SDMT**					
Pre-intervention	76.95 ± 6.40^&^	44.65 ± 10.71^*^	45.55 ± 7.55^*^	95.46	<0.01
Post-intervention	–	57.75 ± 9.75^*^^$^	68.35 ± 11.96^*^, *^#^, ^$^*	19.88	<0.01
**TMT-A**					
Pre-intervention(s)	49.78 (46.03~54.79)^&^	58.28(52.66~77.78)^*^	62.94 (43.61~71.48)^*^	7.89	0.02
Post-intervention(s)	–	57.22 (49.43~75.83)	62.27 (46.39~71.11)	5.45	0.06
**TMT-B**					
Pre-intervention(s)	97.06 ± 21.85^&^	144.62 ± 31.03^*^	149.93 ± 36.26^*^	18.45	<0.01
Post-intervention(s)	–	132.85 ± 31.62^*^	126.86 ± 27.94^*^, *^$^*	9.76	<0.01

* *Compared with the HC group, p < 0.05*.

& *Compared with the CM group pre-intervention, p < 0.05*.

# *Compared with the CM group post-intervention, p < 0.05*.

$ *Compared with the same group pre-intervention, p < 0.05*.

*n, number of cases; HC, healthy control; CM, conventional medical treatment; ER, enriched rehabilitation treatment; MoCA, Montreal Cognitive Assessment; SDMT, Symbol Digit Modalities Test; TMT-A, Trail Making Test part A; TMT-B, Trail Making Test part B*.

*F/χ^2^ and P-values came from a statistical analysis of between-group differences (ER, CM, and HC groups) for continuous variables, and other values of statistical analysis are shown in Supplementary Materials*.

### Serum Glutamate, MDA, and TNF Levels in Patients With PSCI Improved After ER Intervention

**Figure 3:**
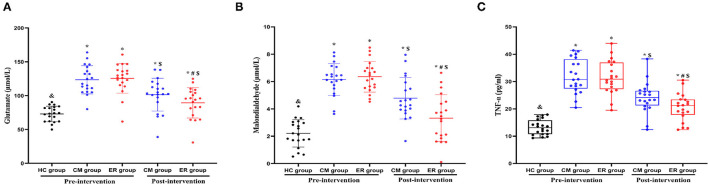
The results of serum indexes in each group pre-intervention and post-intervention. *Compared with the HC group, *p* < 0.05. ^&^Compared with the CM group pre-intervention, *p* < 0.05. ^#^Compared with the CM group post-intervention, *p* < 0.05. ^$^Compared with the same group pre-intervention, *p* < 0.05. HC, healthy control; CM, conventional medical treatment; ER, enriched rehabilitation treatment; MoCA, Montreal Cognitive Assessment; SDMT, Symbol Digit Modalities Test; TMT-A, Trail Making Test part A; TMT-B, Trail Making Test part B. **(A)** Serum glutamate level pre-intervention and post-intervention; **(B)** serum MDA level pre-intervention and post-intervention; **(C)** serum TNF level pre-intervention and post-intervention.

Pre-intervention, serum glutamate, MDA, and TNF levels in patients with PSCI differed significantly (*p* < 0.05) from those in the HC group; however, no differences were detected between the two groups of patients with PSCI (*p* > 0.05) for the above indicators. After the intervention, the serum levels of glutamate, MDA, and TNF in both the PSCI groups were significantly improved compared with the pre-intervention levels (*p* < 0.05). Additionally, the levels of the above indicators in the ER group were significantly better than those in the CM group (*p* < 0.05). However, post-intervention, the serum levels of glutamate, MDA, and TNF in patients with PSCI were still significantly different from those in the HC group (*p* < 0.05) (Table 3 and Figure 3).

**Table 3:** The results of serum indexes in each group pre-intervention and post-intervention.

	**HC group**	**CM group**	**ER group**	**F/χ^2^**	***P***
	**(*n* = 20)**	**(*n* = 20)**	**(*n* = 20)**		
**GLUTAMATE**					
Pre-intervention (μmol/L)	73.02 ±11.35^&^	123.56 ± 20.87^*^	125.54 ± 21.75^*^	51.25	<0.01
Post-intervention (μmol/L)	–	101.46 ± 24.22^*^, *^$^*	86.98 ± 23.29^*^, *^#^, ^$^*	10.02	<0.01
**MALONDIALDEHYDE**					
Pre-intervention (μmol/L)	2.21 ± 1.01^&^	6.15 ± 1.16^*^	6.35 ± 1.12^*^	90.31	<0.01
Post-intervention(μmol/L)	–	4.78 ± 1.52^*^, *^$^*	3.32 ± 1.71^*^, *^#^, ^$^*	15.96	<0.01
**TNF**					
Pre-intervention (pg/ml)	13.12 (10.75~14.89)^&^	30.74 (28.03~37.01)^*^	30.84 (27.76~36.45)^*^	39.35	<0.01
Post-intervention(pg/ml)	–	24.23 (21.77~26.46)^*^, *^$^*	21.24 (18.34~23.37)^*^, *^#^, ^$^*	31.41	<0.01

* *Compared with the HC group, p < 0.05*.

& *Compared with the CM group pre-intervention, p < 0.05*.

# *Compared with the CM group post-intervention, p < 0.05*.

$ *Compared with the same group pre-intervention, p < 0.05*.

*n, number of cases; HC, healthy control; CM, conventional medical treatment; ER, enriched rehabilitation treatment*.

*F/χ^2^ and P-values came from a statistical analysis of between-group differences (ER, CM, and HC groups) for continuous variables, and other values of statistical analysis are shown in Supplementary Materials*.

## Discussion

Enriched rehabilitation represents a novel therapeutic strategy that has been used to enhance neuroplasticity and improve functional outcomes in animal models with central nervous system diseases, including stroke and perinatal hypoxia–ischemia (31, 32). In the present study, we investigated the effect of ER on cognitive function in patients with PSCI as well as the associated underlying mechanisms and provided evidence for the impact of ER in a clinical setting. We found that the serum glutamate level was higher in patients with PSCI than in HCs. Our data further indicated that ER could enhance cognitive function in patients with PSCI, with the results suggesting that this effect may be related to its effect on decreasing serum glutamate levels. In addition, it was found that oxidative stress and inflammation, as indicated by the serum levels of MDA and TNF in patients with PSCI, alleviated after ER intervention.

Post-stroke cognitive impairment is a common complication in stroke survivors that requires complex treatment regimens. ER represents a non-pharmacological therapeutic strategy that combines environmental enrichment with task-oriented exercises, in which a multistimuli environment is accompanied by high-quality social and cognitive exercises. Animal studies have suggested that ER may be a viable approach for treating PSCI (32–34). Additionally, there is some clinical evidence to support that ER exerts a beneficial effect on overall function in stroke patients (9, 35). As expected, in this study, we observed that ER led to a significant improvement (*p* < 0.05) in the overall cognitive function, attentional function, and executive function in patients with PSCI, as determined by the MoCA, SDMT, and TMT scores.

Cognitive function requires the participation of a variety of neurotransmitters, which have to do with synaptic plasticity (36, 37). Glutamate, the major excitatory neurotransmitter in the central nervous system, plays an important role in synaptic plasticity, as well as in learning and memory processes (38, 39). However, an excess of glutamate in the brain can lead to mitochondrial damage and, consequently, the overproduction of reactive oxygen species (ROS) (40, 41). The resulting oxidative stress and inflammation may result in neuronal damage and neurological deficits (42).

The process is as follows: first, excessive glutamate can lead to the overactivation of NMDA receptors in neurons, followed by a massive influx of calcium ions into cells, resulting in mitochondrial damage (43, 44) and the overproduction of ROS (45). Through a series of cascade reactions, these ROS can generate large amounts of peroxides in the brain, such as peroxynitrite (ONOO–), leading to cell death in the central nervous system (12, 46). Second, the glutamate-mediated overactivation of NMDA receptors in neurons can also result in impaired synaptic plasticity and cognitive function (47, 48). Third, other types of receptors for glutamate, such as kainate (KA) and 2-amino-3-hydroxy-5-methyl-4-isoxazole propionic acid (AMPA) receptors, are associated with inflammatory responses (49, 50). The activation of either receptor can promote the release of inflammatory factors. Meanwhile, excessive glutamate can also result in damage to the endothelial cells of the blood–brain barrier, which may allow the invasion of inflammatory factors from the periphery into the central nervous system (51, 52). Hippocampal pyramidal cells are among the most sensitive to the effects of inflammatory factors; accordingly, memory function can be easily impaired under conditions of inflammation (53). Fourth, oxidative stress resulting from increased glutamate levels can interact with and promote post-stroke inflammatory response. Oxidative stress induces the activation of adhesion molecules and promotes the infiltration of immune cells, which, in turn, aggravates the inflammatory reactions in the peripheral immune system and the central nervous system (54).

In addition, under physiological conditions, intracerebral nitric oxide (NO) produced by nNOS mediates communication between nerve cells. However, under inflammatory conditions, iNOS overexpression in cerebral vascular smooth muscle cells and macrophages leads to excessive NO production (55), further aggravating the inflammatory response and contributing to secondary injury after stroke (12). Therefore, it was found that a high glutamate level in the brain tissue and cerebrospinal fluid was associated with poor cognitive outcomes in stroke (13).

Metabolites can be transferred between cerebrospinal fluid and blood through meninges (56, 57). A positive correlation has been established between the level of glutamate in the serum and in the cerebrospinal fluid (11, 58). Therefore, the level of glutamate in the serum can indirectly reflect the level of glutamate in the brain. In our study, we have shown that the serum glutamate level was significantly higher (*p* < 0.05) in patients with stroke than in HCs, which is consistent with previous findings (12). However, we observed that the glutamate level was decreased in both the ER and CM groups after the different interventions (*p* < 0.05). Surprisingly, the serum glutamate level in the ER group was reduced to a greater extent than that in the CM group post-intervention (*p* < 0.05). The above results suggested that the efficacy of ER in enhancing cognitive function in patients with PSCI may be related to its effect on decreasing serum glutamate levels. However, the underlying molecular mechanisms require further investigation.

As previously mentioned, oxidative and inflammatory mechanisms also play a pathogenic role in the process of cognitive impairment post-stroke (12, 54). In this study, we found that the serum levels of MDA, a marker of the oxidative stress response, and TNF, a marker of the inflammatory response, were significantly increased in patients with PSCI compared with those in the HC group. Additionally, in line with the changes observed in the serum glutamate levels, the serum levels of MDA and TNF in the ER group showed a more significant reduction compared with those in the CM group post-intervention (*p* < 0.05). We inferred from the above results that ER can improve cognitive function by abrogating oxidative stress and neuroinflammation induced by increased levels of glutamate post-stroke.

The strengths of our study were that, in addition to the behavioral assessment, we also analyzed serum glutamate levels in patients with PSCI during pre-intervention and post-intervention. Additionally, we investigated oxidative stress- and inflammation-related mechanisms mediated by ER through the measurement of serum MDA and TNF levels. Nonetheless, our study also had several limitations. First, this study was conducted in only one hospital and the sample size was small, which is likely to introduce bias. Additionally, due to the small sample size, we did not stratify participants based on different cognitive levels, which may mask the effect of ER. Furthermore, we could only infer the preliminary mechanisms involved in the effects of ER on PSCI; identification of the precise underlying mechanisms will be explored in a future study.

In conclusion, the present study provided clinical evidence that ER can improve cognitive function in patients with PSCI. The associated mechanism may be related to the negative regulatory effect of ER on serum glutamate, TNF, and MDA levels, which is likely to enhance synaptic plasticity and alleviate oxidative stress- and inflammation-related damage, at least to some extent.

## Data Availability Statement

The original contributions presented in the study are included in the article/Supplementary Material, further inquiries can be directed to the corresponding author/s.

## Ethics Statement

This study was approved by the Medical Ethics Committee of Clinical Medical College of Yangzhou University (Ethical Approval No. 2016055). The patients/participants provided their written informed consent to participate in this study.

## Author Contributions

XW, YP, and JG designed the study and wrote the manuscript. XW, YP, HZ, WD, JuW, JiW, TW, XT, and YL performed the experiments. XW, YP, HZ, WD, JuW, and JG analyzed the data. All authors contributed to the article and approved the submitted version.

## Funding

This work was supported by the National Natural Science Foundation of China (Grant No. 82072533), the Six One Project Scientific Research Project for High-Level Health Talents of Jiangsu Province (Grant Nos. LGY2017028 and LGY2018027), the Key Young Medical Talents in Jiangsu Province (Grant No. QNRC2016339), Yangzhou Science and Technology Development Plan Project (YZ2020201), Huxin fund of Jiangsu Key Laboratory of Zoonosis (Grant No. HX2003), Natural Science Foundation of Shandong Province, China (Grant No. ZR2019MH104), Scientific Research Project of Jiangsu Commission of Health (Grant No. Z2020055), Yangzhou Social Development Funds (Grant No. YZ2021059), the Science Foundation of Subei People's Hospital (Grant No. SBJC21005), and the Science Foundation of Guangzhou First People's Hospital (Grant No. M2019009).

## Conflict of Interest

The authors declare that the research was conducted in the absence of any commercial or financial relationships that could be construed as a potential conflict of interest.

## Publisher's Note

All claims expressed in this article are solely those of the authors and do not necessarily represent those of their affiliated organizations, or those of the publisher, the editors and the reviewers. Any product that may be evaluated in this article, or claim that may be made by its manufacturer, is not guaranteed or endorsed by the publisher.
