# Supplementary material for: The Effects of Enriched Rehabilitation on Cognitive Function and Serum Glutamate Levels Post-stroke
Source: Front Neurol. 2022 Mar 17;13:829090. doi: 10.3389/fneur.2022.829090 (PMC8967952; doi:10.3389/fneur.2022.829090)
Supplement: Supplementary file 1 [file Table_1.DOCX]

MRI examination and calculation of the infarct volume

All patients underwent Diffusion Weighted MRI within 24 hours after admission. The infarct volume (V) was calculated using the formula: V = 0.5 × a × b × c (a is the maximum longitudinal diameter, b is the maximum transverse diameter perpendicular to a, and c is the number of 10 mm slices containing the infarct) by an experienced neurologist who was blinded to the study (Meng et al, 2015).

Exclusion criteria were as follows: ① Multiple stroke history; ② Hypofunction of important organs including heart, lung, liver, and kidney; ③ Patients with severe cognitive and communication disorders; ④ Patients with severe limb dysfunction and inability to move independently; ⑤ Severe cervical spondylosis and cervical instability; ⑥ Patients received thrombolytic therapy with of intravenous tissue plasminogen activator (tPA); ⑦ Patients who were taking drugs that affected plasma glutamate levels (such as glutamine and recombinant GOT).

The sample size was calculated by the following formula:


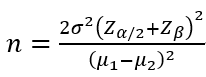


n=2𝜎^2^(Z_α/2_+Zβ)^2^/(μ1-μ2)^2^

Of which, 𝜎 (standard deviation) =2.34, α(bilateral) =0.05，β=0.1，Zα/2=1.96, Zβ =1.28. According to the results from our preliminary experiment and taking MoCA score as the main evaluation index, μ1 was 26.4 and μ2 was 24.2, respectively. As a result, the sample size was calculated out with n=19.87, approximately 20.
